# Increasing Urban Tree Canopy Associated With Reduced Mortality: A Longitudinal Analysis of Chicago Neighborhoods

**DOI:** 10.1029/2025GH001651

**Published:** 2026-08-05

**Authors:** Harrison C. Garcia, Peter M. Graffy, Benjamin W. Barrett, Maxime A. Visa, Jenny Jia, Evan Mallen, Raed Mansour, Graham Briggs, Trent Ford, Donald J. Wuebbles, Norrina Allen, Teresa H. Horton, Abel Kho, Daniel E. Horton

**Affiliations:** ^1^ Feinberg School of Medicine Northwestern University Chicago IL USA; ^2^ Defusing Disasters Working Group Buffett Institute for Global Affairs Northwestern University Evanston IL USA; ^3^ Center for Education in Health Sciences Northwestern University Chicago IL USA; ^4^ Department of Preventive Medicine Northwestern University Chicago IL USA; ^5^ Institute for Public Health and Medicine Northwestern University Chicago IL USA; ^6^ Clinical and Translational Sciences Institute Northwestern University Chicago IL USA; ^7^ Urban Climate Lab Georgia Institute of Technology Atlanta GA USA; ^8^ Metropolitan Planning Council Chicago IL USA; ^9^ Illinois Department of Public Health Chicago IL USA; ^10^ Prairie Research Institute University of Illinois Urbana‐Champaign Champaign IL USA; ^11^ Department of Climate, Meteorology, and Atmospheric Sciences University of Illinois Urbana‐Champaign Urbana IL USA; ^12^ Department of Anthropology Northwestern University Evanston IL USA; ^13^ Department of Earth, Environmental, and Planetary Sciences Northwestern University Evanston IL USA

## Abstract

Urban tree canopy is known to mitigate ambient heat and improve physical and mental health but the longitudinal interactions and impacts of tree canopy, temperature, and mortality are not well understood. Using high resolution tree canopy estimates, temperature, mortality, and demographic data from 2011 to 2021, we implemented negative binomial generalized estimating equations to model all‐cause and disease‐specific mortality based on tree canopy cover, year‐to‐year changes in tree canopy cover, and maximum temperature, adjusting for area‐level demographics in Chicago, IL. We used K‐means clustering to identify patterns of canopy, temperature, and mortality disparities across Chicago community areas. There were 220,711 decedents during the study period (115,974 male, 104,734 female; mean age at death: 69.4 ± 19.9 years). Existing canopy coverage was not statistically significantly associated with mortality (incidence rate ratio [IRR] = 0.995, 95% confidence interval [CI] = 0.984–1.007). However, each year‐to‐year percent increase in canopy was associated with around a 10% reduction in mortality (IRR = 0.902, [0.871–0.935]) with significant associations for cause‐specific cardiovascular (IRR = 0.908, [0.869–0.948]), mental health (IRR = 0.836, [0.784–0.892]), musculoskeletal (IRR = 0.907, [0.832–0.989]), and respiratory (IRR = 0.887, [0.833–0.945]) diseases. Cluster analysis identified that neighborhoods on Chicago's South and West sides were characterized by high temperatures, greater absolute canopy loss despite elevated baseline canopy levels, and increased mortality. Among the hottest neighborhoods, canopy‐temperature interaction models demonstrated that areas experiencing larger year‐to‐year canopy losses had higher yearly mortality. Protecting tree canopy cover from losses over time, particularly in vulnerable areas with higher temperatures, may present a key opportunity to reduce mortality and mitigate impacts of climate change.

## Plain Language Summary

Trees in cities do more than provide shade, they can also protect health. Many studies have looked at how one measurement of tree canopy cover relates to health. But few have examined how year‐to‐year changes in tree canopy affect mortality over time. We studied Chicago from 2011 to 2021 to see how tree canopy cover, summer heat, and deaths were connected. Using data on temperature, tree canopy cover, demographics, and mortality, we found that neighborhoods with increasing year‐to‐year tree canopy had lower associated death rates. We also found that these increases in tree canopy cover were associated with less risk of dying from heart, lung, mental health, and musculoskeletal diseases. In addition, we found that within the hottest neighborhoods, which clustered on Chicago's South and West sides, mortality increased more as year‐to‐year tree canopy losses increased. Therefore, planting and maintaining trees, particularly in hotter neighborhoods, could lower mortality and make communities more resilient to rising temperatures.

## Key Points

- Total canopy cover was not associated with mortality, but year‐to‐year increases were linked to lower all cause and cause specific mortality
- Year‐to‐year tree canopy loss is significantly associated with increased mortality, particularly in the hottest neighborhoods
- Effects exist regardless of baseline canopy, highlighting the need to preserve existing canopy in addition to planting

## 1 Introduction

Trees have long represented life and resilience across various cultures (Rival, 1998). Although most of the world's population lives in urban areas due to socioeconomic benefits, urbanization has created disparities in access and exposure to trees. These inequities have been further entrenched by income inequality and residential segregation (Gerrish & Watkins, 2018; Swope et al., 2022). Research consistently shows that proximity to trees and greenness is associated with numerous health benefits, such as reduced cardiometabolic (Astell‐Burt & Feng, 2020), respiratory (Lovasi et al., 2008), and overall mortality (Wolf et al., 2020). Several causal mechanisms for the benefits of green space on human health have been studied, from improving mental health on the individual level to mitigating pollution and encouraging a healthy lifestyle on a population level (Markevych et al., 2017).

Climate change, driven by anthropogenic changes in land cover and rising greenhouse gas concentrations in the atmosphere, has caused global temperatures to increase, particularly in urban areas due to the urban heat island effect (Wuebbles et al., 2017), with notable consequences for human health (Graffy et al., 2026). Extreme heat has caused more deaths in the United States (U.S.) than all other weather‐related disasters since 2002 (Weather Related Fatality and Injury Statistics: 80‐Year List of Severe Weather Fatalities, 2024).

Unlike general vegetation and greenery, trees uniquely mitigate urban heat (Mallen et al., 2020; Mcdonald et al., 2024). Reduced tree canopy has previously been linked to elevated levels of heat related morbidity and mortality (Mcdonald et al., 2024). Even the presence of forest pests like the emerald ash borer, responsible for the loss of 100 million trees in the U.S., has been tied to elevated cardiovascular and respiratory‐related mortality (Donovan et al., 2013). By cooling cities and reducing stress, increased tree canopy may reduce crime and increase social cohesion (Wolf et al., 2020). Additionally, trees can be counted, planted, and tracked, making trees a more tangible marker for actionable public health interventions. Accordingly, public policy efforts in the U.S. and globally in cities like Paris, London, and Shanghai specifically prioritize tree planting to enhance urban resilience and health.

Chicago, Illinois, the third largest city in the U.S., serves as a powerful setting to study tree canopy, temperature, and mortality over time. Consistent with broader trends over the past century, the Chicago region has experienced significant warming, with temperatures rising more in recent decades. The average daily temperature from 2002 to 2021 increased ∼1°C in this region relative to a 1901–1960 reference period (Wuebbles et al., 2021). The 1995 Chicago heat wave, which resulted in over 700 deaths, remains one of the most prominent historical examples of how extreme heat disproportionately affects vulnerable populations (Whitman et al., 1997). Given that 11 of the world's 15 largest cities are located on the shores of major water bodies and ∼50% of the global population lives near freshwater, Chicago, situated on the southwest shore of Lake Michigan, also exemplifies how proximity to water can cool the surrounding climate (Kummu et al., 2011).

Chicago is also home to one of the largest urban conservation efforts in the U.S.—the Forest Preserve District of Cook County (Chicago Urban Forest Management Plan, 2023). Spanning approximately 70,000 acres, these protected green spaces run along the border of the city's South and West sides, standing in contrast to the historically low investment within these neighborhoods. The stark differences between neighborhood environments in Chicago have been driven by a history of redlining that has concentrated well maintained, high quality green space in wealthier, non‐Hispanic White areas (Stuhlmacher & Kim, 2024). This phenomenon of segregation in urban planning is not exclusive to Chicago or the U.S., as cities worldwide, whether by design or circumstance, exhibit similar patterns (Newman & Thornley, 2011).

While the association between canopy and morbidity is important, studying mortality may be most consequential for human health, healthcare costs, and public health policy (Pol & Thomas, 1992). Most previous studies that have investigated the relationship between urban temperature, tree canopy, and mortality have used cross‐sectional methods that only take 1 year of tree canopy into account (Astell‐Burt & Feng, 2020; Keralis et al., 2020; Knobel et al., 2021; Lovasi et al., 2008; Sinha et al., 2022; Ulmer et al., 2016; Wei et al., 2024). Studies have found that tree planting is associated with significant reductions in mortality (Donovan et al., 2022), but the simultaneous growth and death of tree canopy, its year‐to‐year changes, and its interaction with temperature remain unexplored.

This study longitudinally examines how temporal shifts in tree canopy coverage relate to urban heat and mortality outcomes. While determining citywide associations between tree cover and mortality is valuable, it is equally critical to identify specific neighborhoods where increasing or maintaining canopy could address disparities in heat‐related mortality. The aims of this study are therefore twofold: (a) quantify the association between tree canopy cover, temperature, and mortality across Chicago over time, and (b) identify neighborhoods impacted by canopy, temperature, and mortality disparities to inform targeted interventions.

## 2 Materials and Methods

### 2.1 Data Sources

The U.S. Department of Agriculture (USDA) Forest Service's National Land Cover Database (NLCD) provided annual tree canopy cover (TCC) maps (version 2021.4) from 2011 to 2021 at 30‐m spatial resolution (Dewitz, 2023). In this study, TCC is defined as the proportion of land area covered by the vertically projected crowns of trees. The NLCD maps capture TCC as a continuous percent calculated by models built from Landsat and Sentinel‐2 imagery calibrated with field‐interpreted reference data and further adjusted to filter out shrubs or other non‐tree vegetation. We used ArcPy with ArcGIS Pro by Esri to calculate the average percent of land cover comprised of tree canopy for each Chicago census tract across all years. Census tracts represent U.S. Census Bureau geographical areas with average populations of ∼4,000 residents (United States Census, 2024). Daily temperature estimates from Daymet V4 (Oak Ridge National Laboratory), which provides statistically interpolated air temperatures at a 1 km^2^ spatial resolution, were aggregated at the census tract level (Thornton et al., 2022). Average maximum (max) temperature for summer months (June–August) was computed from daily estimates for each year. To adjust for environmental co‐exposures that might be confounders, that is, air pollution, we also included annual NO_2_ concentrations for each census tract. Census tract‐level NO_2_ was obtained by averaging 1‐km gridded surface concentrations across tract boundaries (Anenberg et al., 2022).

Complete citywide mortality records were obtained from the Illinois and Chicago Departments of Public Health. All analyses of the mortality records adhered to a Data Use Agreement executed between the Illinois and Chicago Departments of Public Health. Mortality records were aggregated by year and by census tract of the decedent's residential address. Each record also contained the associated causes of death as denoted by International Classification of Diseases, 10th Revision (ICD‐10) codes (International Statistical Classification of Diseases and Related Health Problems 10th Revision, 2019). We defined all‐cause mortality as all deaths recorded in Chicago during the study period.

Demographic data were obtained from the U.S. Census Bureau's American Community Survey (ACS) 5‐Year Estimates and were accessed using the IPUMS and TidyCensus packages in R version 4.4.0 (United States Census, 2024). Selected variables included census tract‐level total population, median income, median age, and census designated race/ethnicity. We also included seven Chicago regions (North, Northwest, West, Central, South, Southwest, and Far South) indicating where each census tract is located to control for unmeasured area‐level confounding, such as broader spatial and policy‐level differences (“City of Chicago Regions and Community Areas”, 2010). All variables were available for all study period years.

Race and ethnicity were not directly included as covariates in our analysis. Past literature has demonstrated that race, as a social construct, correlates with, but is not causative of, disease or mortality (Williams et al., 2016). Rather, disparities are more accurately explained by socioeconomic factors such as access to healthcare, income, education, and neighborhood‐built environments. Instead, we computed tract‐level racial and ethnic population heterogeneity using Shannon entropy (Shannon, 1948) to control for broader effects of segregation using this equation:

$$H=-\sum_{i=1}^{4}p_{i}\;\ln\left(p_{i}\right)$$

where *H* represents the entropy (diversity) of racial/ethnic composition within a census tract–year, *i* indexes the four census designated categories of White, Black, Hispanic/Latino, and Asian, and *p*_*i*_ is the proportion of the tract population belonging to group *i*. Higher values of *H* indicate greater racial/ethnic heterogeneity, while values closer to zero indicate greater homogeneity.

### 2.2 Statistical Methods

Data cleaning was done in Python 3.12, while statistical models and figures were made using R 4.4.0. To evaluate the association between tree canopy and mortality over time, we employed generalized estimating equations (GEE) using the geeM R package with a negative binomial distribution to account for overdispersion and within‐tract correlation over time. All models included an offset of the log of total yearly population to account for variation in tract population size.

To test whether TCC is associated with all‐cause mortality, we constructed two primary models with canopy coverage as the exposure: one examining the percent of land covered by tree canopy, and another assessing year‐to‐year changes in TCC. Year‐to‐year changes in TCC were calculated by subtracting the percent canopy cover of the preceding year from that of the current year. Citywide results were reported in incident rate ratios (IRRs) with 95% confidence intervals which represent population‐averaged effects across all census tracts for a given model. The effect size unit for IRRs represent associated mortality changes per every 1% of TCC or 1% year‐to‐year TCC changes. All models included yearly census tract‐level statistics on average max summer temperature, median income, ethnic/racial Shannon entropy, NO_2_ concentrations, Chicago region, and a population log offset as controls.

### 2.3 Sensitivity Analyses

TCC models were rerun excluding data from 2020 onward to assess the potential impact of the COVID‐19 pandemic. Sensitivity models using Google's Tree Equity Score (TES) canopy estimates and the Normalized Difference Vegetation Index (NDVI) were computed to assess the validity of using NLCD TCC maps in our models (Vermote, 2019). To assess the completeness of our model specification, we ran a model also including unemployment, educational attainment, and insurance coverage from ACS data.

### 2.4 Tree Canopy and Temperature Interactions

To investigate how temperature modifies the relationship between tree canopy and mortality (and vice‐versa), we developed two additional models using the same control variables listed in Section 2.2 and incorporating interaction terms between canopy coverage and maximum temperature. We compared linear and polynomial specifications of the interaction term to assess potential nonlinearity of the models. Model fit was evaluated using the quasi‐likelihood under the independence model criterion (QIC) from the MuMIn R package. Lower QIC values were interpreted as indicating improved model fit. The polynomial specification, which had a superior fit, was used in our final interaction models. We also ran a sensitivity model using splines to better assess nonlinearity when plotting the tree canopy‐temperature interactions. The interaction modeling approach helps determine the relationship between both overall canopy cover and changes in canopy cover with mortality, while assessing whether temperature influences the strength of these associations. Since nonlinear interaction models can be difficult to interpret directly from model coefficients, we visualized the predicted effects by plotting each interaction model with temperature held constant at values from five arbitrary but representative percentiles (5th, 25th, 50th, 75th, and 95th) for both existing tree canopy and yearly changes in canopy cover as described in the following formula:

$$\log\left(E\left(Y\right)\right)=\beta_{0}+\beta_{1}\left(\text{Canopy}\right)+\beta_{2}\left(\text{Temperature}\right)+\beta_{3}\left(\text{Canopy}\;*\;\text{Temperature}\right)+\beta_{4}{\left(\text{Canopy}\;*\;\text{Temperature}\right)}^{2}+Z+\log\left(\text{Population}\right)$$

where log(*E*(*Y*)) represents the natural logarithm of the expected mortality count, consistent with the log link function of the negative binomial model, *β*_1_ and *β*_2_ represent the main effects of tree canopy and temperature, *β*_3_ and *β*_4_ capture the linear and quadratic components of their interaction, *Z* denotes the vector of tract‐level covariates, and log(*Population*) is the offset that scales mortality counts by tract population. Predicted mortality rates per 100,000 residents were plotted across the observed range of canopy and yearly canopy change while holding all other covariates at their mean values.

### 2.5 Disease‐Specific Mortality Associations

We also examined the association of tree canopy with mortality from health conditions that are known to be modified by physical activity or behavior‐linked factors. Mortality counts were subset into four major categories of diseases contributing to death: cardiovascular, respiratory, mental health/psychiatric, and musculoskeletal from ICD‐10 codes. Each category was defined by selecting death records with ICD‐10 codes beginning with the following letters: “I” for cardiovascular diseases, “J” for respiratory diseases, “F” for mental health and psychiatric disorders, and “M” for musculoskeletal conditions. A refined analysis was conducted focusing on musculoskeletal conditions with ICD‐10 prefixes related to conditions for which prior literature notes that the mortality risk may be lessened through physical activity: “M15‐M19” for osteoarthritis (Kraus et al., 2019), “M05‐M06” for rheumatoid arthritis, “M80‐M81” for osteoporosis (Howe et al., 2011), “M65,” “M70,” and “M75‐M77” for soft tissue disorders (Cooper et al., 2023), as well as “S” codes for fractures and injury‐related conditions (Gregg et al., 2000). GEE models were run separately for each cause of death subset using the same modeling framework as the all‐cause mortality analysis.

### 2.6 Community Area Level Estimates

To explore spatial patterns and identify neighborhood‐level disparities, we aggregated the 801 Chicago census tracts into their respective 77 community areas (“Chicago's Neighborhoods: The 77 Community Areas and Their History”, 2025). Community areas are officially recognized divisions of Chicago defined in the 1920s and used for various urban planning applications.

### 2.7 K‐Means Clustering Analyses

We performed K‐means clustering analyses using the R cluster package to explore whether distinct patterns of tree canopy, temperature, and mortality occur geographically across Chicago's community areas, emulating the methods employed by Graffy et al. (2026). Data were averaged across all years from 2011 to 2021 per community area. All‐cause mortality counts were divided by community area population and multiplied by 100,000 to create mortality rates per 100,000 residents. A 3‐D cluster analysis was conducted using mortality rates, average TCC, and average maximum summer temperature. The optimal number of clusters was identified using the Elbow Method. This approach allowed us to classify community areas based on shared environmental and health characteristics, offering a more nuanced understanding of how tree canopy, heat, and mortality measures are distributed across Chicago, and where tree planting initiatives may be most effective.

### 2.8 Hypothetical Tree Planting‐Associated Annual Mortality Reductions

To investigate how increasing annual tree canopy could relate to reductions in mortality, we first estimated the current number of trees per community area (averaged over 2011–2021) as the quotient of TCC in square meters and the average area occupied by a tree: 55 m^2^. While this estimate has been validated previously by the USDA Forest Service for the use in the i‐Tree Canopy tool and the American Forests Tree Equity Score (Tree Equity Score: Methods and Data [Dataset], 2024), we decided to scale these estimates using the total citywide tree count from Morton Arboretum's 2020 Chicago Region Tree Census to best align with observed data (2020 Chicago Region Tree Census Report, 2020). To provide an interpretable measure of the potential scale and impact of a tree planting initiative, we then applied a hypothetical city‐wide 0.03% increase in yearly canopy cover, which is roughly equivalent to the number of trees the City of Chicago has planted per year in the Our Roots Chicago tree planting initiative, a five year plan to plant 75,000 new trees beginning in 2022 (“Mayor Lightfoot Announces New Tree Equity Initiative ‘Our Roots Chicago’”, 2022). The required new trees needed to increase canopy cover by 0.03% was calculated with the same methods as previously described. The mortality reduction associated with the hypothetical 0.03% year‐to‐year increase in TCC was then estimated using the regression coefficients from the year‐to‐year TCC negative binomial GEE model.

## 3 Results

### 3.1 Population Overview

There were 220,711 decedents during the study period, including 104,734 females (47.5%) and 115,974 males (52.5%). The average age at death was 69.4 years (standard deviation: 19.9). Descriptive statistics for the decedents, including age, sex, and other relevant variables, are detailed in Table 1.

**Table 1:** Descriptive Statistics of All Deaths in Chicago From 2011 to 2021

Age (years), mean (SD)	69.4 (19.9)
Sex, *n* (%)	
Female	104,734 (47.5)
Male	115,974 (52.5)
Unknown	3 (0.0)
Race/ethnicity, *n* (%)	
Asian	7,259 (3.3)
Black	107,409 (48.7)
Hispanic	28,408 (12.9)
Other	2,859 (1.3)
White	74,776 (33.9)
Education, *n* (%)	
8th grade or less	33,398 (15.1)
9th–12th grade, No diploma	24,499 (11.1)
High school graduate or GED	84,938 (38.5)
Some college, No degree	25,967 (11.8)
Associate degree	12,121 (5.5)
Bachelor's degree	18,612 (8.4)
Master's degree	8,443 (3.8)
Doctorate	3,509 (1.6)
Unknown	9,224 (4.2)
Marital status, *n* (%)	
Divorced	33,281 (15.1)
Married/partnered	59,976 (27.1)
Never married	61,053 (27.7)
Unknown	4,191 (1.9)
Widowed	62,210 (28.2)
Manner of death, *n* (%)	
Accident	12,744 (5.8)
Homicide	6,189 (2.8)
Natural	197,902 (89.7)
Suicide	2,249 (1.0)
Unknown^a^	1,627 (0.7)

^a^ “Unknown” includes deaths classified as could not be determined (*n* = 1,121), not specified (*n* = 308), and pending investigation (*n* = 198). SD = Standard deviation.

**Figure 1:**
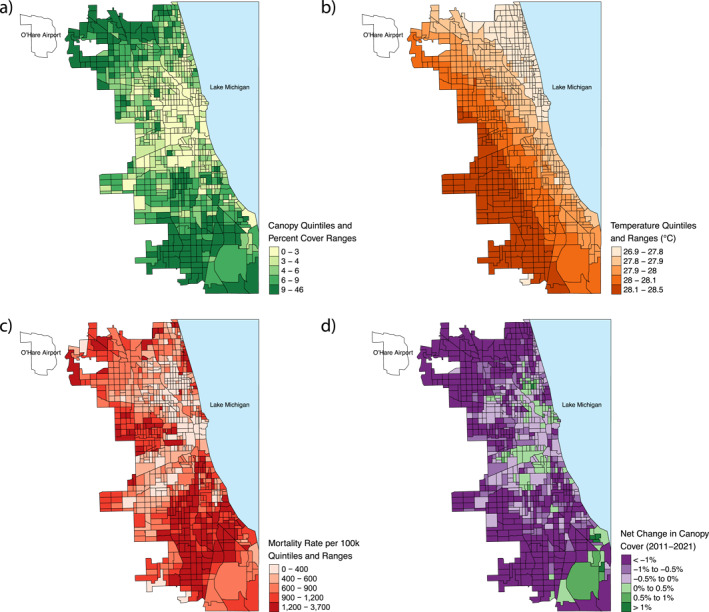
Census tract‐level environmental and health factors in Chicago (2011–2021) including (a) quintiles of tree canopy percent land area coverage (averaged over 2011–2021), (b) quintiles of maximum summer temperature (°C, averaged over 2011–2021), (c) quintiles of annual mortality rate per 100,000 people (averaged over 2011–2021), and (d) quintiles of net change in tree canopy percent land area coverage from 2011 to 2021.

Figures 1a–1d shows quintile maps of tree canopy cover, maximum temperature, mortality rate per 100 k (averaged across all years), and net canopy change from 2011 to 2021 at the census tract level. Plotting NLCD TCC by community area (Figure S1 in Supporting Information S1) demonstrates similarities with existing canopy estimate maps from the City of Chicago and the Morton Arboretum (“Chicago Region Trees Initiative”, 2024; Chicago Urban Forest Management Plan, 2023).

### 3.2 Longitudinal Associations and Interactions of Tree Canopy, Temperature, and Mortality

Negative binomial GEE models found that TCC across Chicago census tracts had a statistically insignificant association with all‐cause mortality (IRR = 0.995, 95% confidence interval [CI]: 0.984–1.007) (Table 2). However, year‐to‐year increases in tree canopy were associated with a statistically significant reduction in mortality (IRR = 0.902 95% CI: 0.871–0.935).

**Table 2:** Negative Binomial Generalized Estimating Equations Model Results

Mortality cause	Model^a^	IRR	95% CI lower	95% CI upper
All Cause	Tree Canopy Cover	0.995	0.984	1.007
All Cause	Year‐to‐year Change in Tree Canopy Cover	0.902	0.871	0.935
Cardiovascular	Tree Canopy Cover	1.002	0.991	1.014
Cardiovascular	Year‐to‐year Change in Tree Canopy Cover	0.908	0.869	0.948
Mental Health	Tree Canopy Cover	1.014	1.005	1.023
Mental Health	Year‐to‐year Change in Tree Canopy Cover	0.836	0.784	0.892
MSK	Tree Canopy Cover	1.004	0.995	1.014
MSK	Year‐to‐year Change in Tree Canopy Cover	1.015	0.912	1.130
MSK Subset	Tree Canopy Cover	1.003	0.996	1.010
MSK Subset	Year‐to‐year Change in Tree Canopy Cover	0.907	0.832	0.989
Respiratory	Tree Canopy Cover	1.006	0.997	1.015
Respiratory	Year‐to‐year Change in Tree Canopy Cover	0.887	0.833	0.945

*Note.* CI, Confidence interval; IRR, Incidence rate ratio; MSK, Musculoskeletal.

^a^ Models included census tract‐level statistics on average max summer temperature, median income, ethnic/racial entropy, NO_2_ concentrations, Chicago region, and a population log offset as controls.

**Figure 2:**
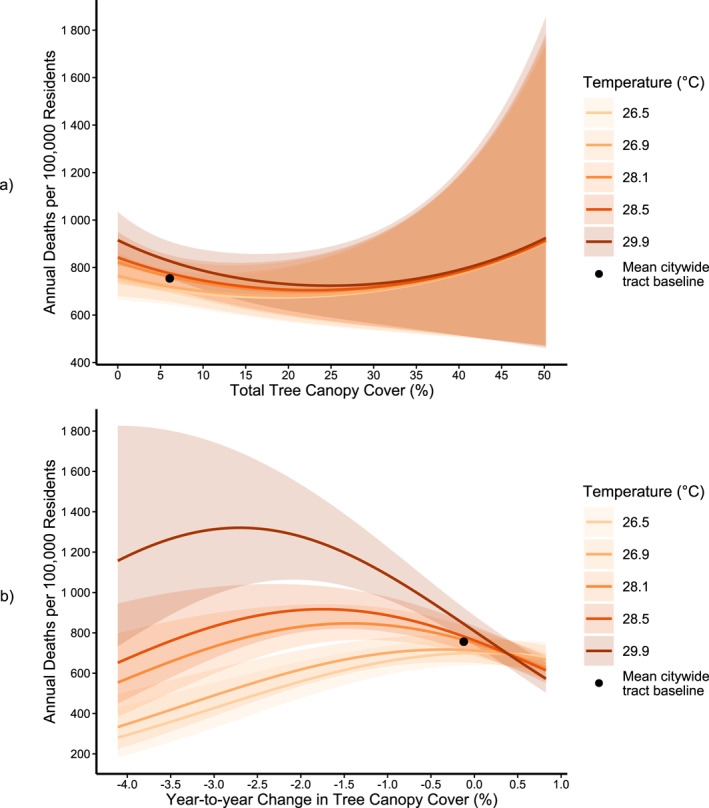
Polynomial interaction effects of tree canopy cover and temperature on mortality. Estimates were derived from negative binomial generalized estimating equation models adjusting for census tract‐level statistics on average max summer temperature, median income, ethnic/racial entropy, NO_2_ concentrations, Chicago region, and a population log offset as controls. (a) Interaction between total tree canopy cover (%) and average maximum temperature on mortality rate; results from this model were statistically insignificant. (b) Interaction between year‐to‐year percent change in tree canopy cover and average maximum temperature on mortality rate; results from this model were statistically significant.

Significant interaction between year‐to‐year changes in canopy cover and temperature were observed. Like the all‐cause TCC model, the polynomial interaction model between TCC and temperature was statistically insignificant. We still present the interaction effects for this model at different temperature percentiles (5th, 25th, 50th, 75th, and 95th percentiles, corresponding to 26.5°C, 26.9°C, 28.1°C, 28.5°C, and 29.9°C, respectively) which should be interpreted with caution. Plotting demonstrated a U‐shaped pattern for total TCC, with predicted deaths decreasing as total TCC increased up to around 20%–25% total TCC (Figure 2a). As total TCC increased beyond this point, predicted deaths increased. This pattern was consistent across temperatures, with higher temperature tracts having a higher predicted death count at lower total TCC (left‐hand side of the U‐shape), and a later turning point in the U‐shape. Modeling using splines also demonstrated increasing mortality at higher TCC values (Figure S2 in Supporting Information S1). Our year‐to‐year change in TCC and temperature polynomial interaction model demonstrated statistical significance (Figure 2b). As losses to year‐to‐year TCC decreased, annual deaths were predicted to increase and then decline. This decline in predicted deaths was most pronounced among tracts at the 95th temperature percentile, with fewer losses in TCC over time having a strong association with reduced predicted death count. In the lowest temperature tracts, annual deaths were predicted to modestly increase along with fewer losses in year‐to‐year TCC up to around 0% change in year‐to‐year TCC, where predicted deaths then plateaued.

### 3.3 Sensitivity Analyses

Specific results for each sensitivity model can be found in Supporting Information S1. Excluding the COVID‐era years (2020 onward) revealed higher annual NLCD TCC had a slight positive association with mortality, whereas year‐to‐year changes remained of similar statistical significance and direction to our main models. In models substituting Google TES TCC data for the NLCD TCC measurements, we observed a small but statistically significant positive association between TCC and mortality. Pre‐COVID, higher NDVI was statistically significantly associated with higher mortality, whereas year‐to‐year increases in NDVI were statistically significantly associated with lower mortality. NDVI models including all years demonstrated associations with very small effect sizes between total or year‐to‐year changes in greenness and mortality. The statistical significance and direction of effect estimates did not change with the addition of unemployment, educational attainment, and insurance coverage from ACS data.

### 3.4 Disease‐Specific Mortality Associations

Disease‐specific mortality outcomes are listed in Table 2. Counts of ICD prefix disease types can be found in Table S1 in Supporting Information S1. Year‐to‐year increases in TCC (IRR = 0.908, 95% CI: 0.869–0.948) were associated with reductions in cardiovascular‐related mortality. Mental health‐related mortality was also reduced in association with increasing yearly TCC (IRR = 0.836; 95% CI: 0.784–0.892). Total TCC, however, was statistically significantly associated with increased mental health‐related mortality (IRR = 1.014; 95% CI: 1.005–1.023). In the general analysis, no statistically significant associations were observed with MSK‐related mortality. However, a subset of MSK conditions modifiable by physical activity (e.g., osteoarthritis, rheumatoid arthritis, osteoporosis, fractures) revealed a statistically significant protective association with increasing year‐to‐year TCC (IRR = 0.907, 95% CI: 0.832–0.989). Increasing year‐to‐year TCC was associated with a statistically significant reduction in respiratory‐related mortality (IRR = 0.887, 95% CI: 0.833–0.945).

### 3.5 K‐Means Clustering Analyses

**Figure 3:**
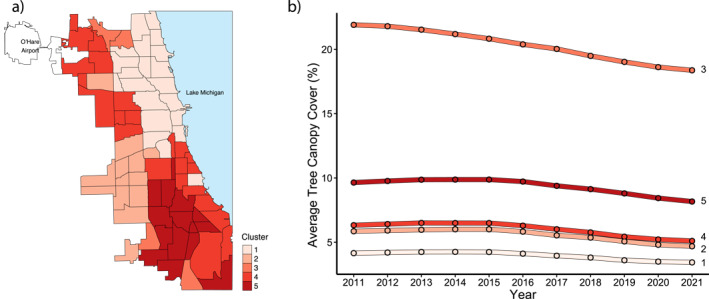
Community area clusters and time series of canopy change. (a) 3‐D K‐Means clustering of Chicago community areas by mortality, canopy, and temperature, with clusters ordered by increasing mortality (1 = lowest, 5 = highest). (b) Time series plot of average tree canopy cover (%) by cluster from 2011 to 2021. Absolute change in canopy cover from 2011 to 2021 for Clusters 1–5: −0.7%, −1.2%, −3.5%, −1.2%, −1.5%. Relative change in canopy cover from 2011 to 2021 for Clusters 1–5: −17.3%, −19.9%, −16.1%, −19.3%, −15.3%.

Five distinct community area clusters of tree canopy, temperature, and mortality were identified. Sociodemographics for each cluster are detailed in Table 3, while Figure 3a shows the clusters mapped across Chicago, revealing notable spatial autocorrelation. Figure 3b shows a time series plot by cluster of average TCC for each year.

**Table 3:** K‐Means Cluster Sociodemographics With Estimated Mortality Reduction From Tree Planting

Cluster	1	2	3	4	5
Average Mortality Rate per 100 k	514	610	785	966	1301
Average Median Income	$75,018.73	$49,491.46	$77,825.37	$40,826.35	$37,364.93
Average Median Age (years)	34	33	40	35	39
Average Total Population	1,107,205	489,652	87,353	699,467	338,740
Percent White	67%	50%	65%	28%	6%
Percent Black	11%	12%	19%	57%	90%
Percent Asian	10%	3%	9%	4%	1%
Percent Hispanic	24%	68%	12%	21%	4%
Racial/ethnic Entropy	0.87	0.81	0.70	0.58	0.24
Percent with Bachelor's Degree or Higher	66%	21%	50%	28%	24%
Percent Unemployed	7%	12%	9%	16%	23%
Percent Insured	99%	99%	99%	98%	99%
Average Maximum Temperature (°C)	27.8	28.1	28.0	27.9	28.0
Average Nitrogen Dioxide NO_2_ (ppb)	17.9	16.7	16.1	16.6	16.1
Average Canopy Cover (%)	4.1	5.9	20.2	6.3	9.5
Relative Change in Canopy from 2011 to 2021 (%)	−17%	−20%	−16%	−19%	−15%
Total Current Trees	456,564	471,739	1,211,262	993,839	863,594
Additional Trees for +0.03% Cover	3,410	2,388	1,590	4,198	2,619
Total Mortality Reduction w/(95% CI)	28 (27–29)	17 (16–18)	5 (5–5)	32 (31–33)	23 (22–24)

Cluster 1, with the lowest average mortality rate, is predominantly located in the Central/North regions of Chicago, and is characterized by high socioeconomic status, including high income and education levels. Despite these advantages, it has the lowest average TCC (driven by the inclusion of downtown Chicago in this cluster) but also the lowest average temperature as mediated by lakefront proximity. Cluster 2 is located on the West side of Chicago and has a diverse racial composition, majority Hispanic residents, moderate income, younger median age, low average TCC, and moderate mortality rates. Cluster 3 is spatially disconnected and includes neighborhoods in the Northwest and Far South regions near the Forest Preserve of Cook County, with the highest average TCC, a predominantly White population, and high median income. This cluster demonstrated relatively lower mortality rates compared to Clusters 4 and 5. Cluster 4 is similarly spatially disconnected, including neighborhoods in regions spreading from the South/Southeast of Chicago along Lake Michigan to the West/Northwest. Cluster 4 is characterized by relatively low average TCC, moderate socioeconomic indicators, high segregation and high mortality. Cluster 5 had the highest average mortality rate and was primarily located on the Southwest/Far Southwest sides of Chicago. Cluster 5 is characterized by a low median income, a predominantly Black population with the highest segregation by Shannon entropy, higher average TCC, and high unemployment.

We generated a time series plot of average TCC by cluster to assess how areas with shared environmental and health profiles each experience canopy changes over time. Clusters demonstrated similar directional trajectories—an overall decrease in average TCC from 2011 to 2021. Clusters 5 and 3 experienced the largest absolute decreases in canopy cover (−1.5% and −3.5%, respectively), while Clusters 1, 2, and 4 experienced larger relative percent decreases in canopy cover over time (−17.3%, −19.9%, and −19.3% respectively), indicating more dramatic proportional losses despite lower baseline canopy (Figure 3b).

### 3.6 Hypothetical Tree Planting‐Associated Annual Mortality Reductions

**Figure 4:**
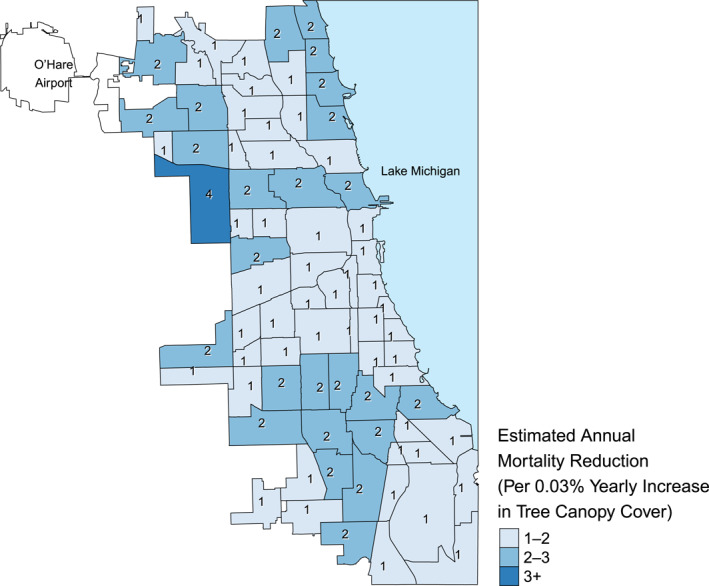
Community area‐level estimates for number of annual reduced deaths associated with a hypothetical constant 0.03% increase in city‐wide year‐to‐year tree canopy cover (from a baseline of 0% year‐to‐year change). This map shows estimated reductions in yearly mortality by Chicago community area, based on results from our year‐to‐year TCC negative binomial GEE model. Census tract estimates were summed into their respective community areas. The predicted mortality reduction represents the effect of a sustained 0.03% annual increase in canopy cover, corresponding to the planting of approximately 14,000 trees in the first year, a number roughly equivalent to Chicago's current yearly tree planting efforts.

A hypothetical 0.03% increase in year‐to‐year TCC was calculated to be equivalent to planting ∼14,000 trees across the city. Planting these trees was found to be associated with 105 (95% CI: 101–109) fewer deaths per year. Tree counts required to increase year‐to‐year TCC by 0.03% and resultant mortality reductions within the five clusters are provided in Table 3. Associated mortality reductions for a 0.03% increase in year‐to‐year TCC were calculated and mapped by community area across the city (Figure 4).

## 4 Discussion

Our study demonstrated a statistically significant association between increasing TCC over time and reduced mortality in Chicago. We found reductions for all‐cause, cardiovascular, respiratory, mental health, and MSK‐related deaths. Specifically, each 0.03% increase in year‐to‐year TCC was associated with 105 (95% CI: 101–109) fewer deaths per year. Our interaction models suggest that the association between increasing TCC and mortality is not constant across temperatures and varies in a nonlinear fashion. We observed a strong protective effect on mortality among the hottest census tracts as year‐to‐year losses in TCC declined. This suggests that protecting and maintaining existing canopy may yield even higher benefits than tree planting alone. As temperatures continue to rise, increasing urban tree canopy will play an essential role in mitigating heat stress, particularly in communities most vulnerable to climate change. Additionally, we applied 3‐D K‐means clustering to Chicago community areas to identify which populations are most affected by mortality, temperature, and tree canopy disparities. This method identified specific neighborhoods where interventions such as tree planting and maintenance could most effectively reduce temperature‐related mortality and enhance urban resilience.

Our results also suggest that expanding tree canopy may reduce the risk of mortality from chronic diseases. Particularly notable are the associations found between increasing year‐to‐year TCC and reduced rates of cardiovascular, exercise‐modifiable MSK conditions, and respiratory‐related mortality, which are known to be modified by physical activity (Wolf et al., 2020). Paradoxically, higher annual TCC was found to have a statistically significant association with increased mental health‐related mortality. This may be due in part to the fact that substance use disorders, the most common mental health‐related ICD‐10 code in this subset (Table S1 in Supporting Information S1), are known to have higher prevalence among more socioeconomically disadvantaged areas, which our cluster analysis found to be characterized by higher baseline TCC (Baptiste‐Roberts & Hossain, 2018). However, mental health‐related mortality had the strongest effect size among our cause‐specific year‐to‐year TCC change models. This finding does align with previous literature proposing that improved mental health is one of the main mechanisms by which TCC influences morbidity and mortality (Markevych et al., 2017). Our finding that respiratory‐related mortality has a strong associated reduction with year‐to‐year increasing TCC aligns with prior evidence suggesting that the air‐quality benefits of greater tree canopy confer a net protective effect on respiratory health despite higher pollen exposure (Lovasi et al., 2008).

Our 3‐D K‐means cluster analysis found that Cluster 1, comprised of Downtown Chicago and North Side community areas, had the lowest average TCC (4%), highest NO_2_ concentrations, and the lowest average mortality rate. This finding highlights how other factors, such as socioeconomic resources, access to healthcare, the built environment, social cohesion, and proximity to the cooling effects of water all contribute to heat‐related mortality rate disparities within distinct neighborhoods and communities. Although statistically insignificant, plotting the interaction between TCC and temperature demonstrated increases in mortality above TCC of ∼20%. Community areas with these canopy values grouped together in Cluster 3, which was also characterized by the lowest population and highest age. As shown in Figure S3 in Supporting Information S1, there are relatively few tract‐years with canopy values above 20.2%, the average TCC for Cluster 3, similar to the turning point observed in Figure 2a. This pattern suggests that the apparent increase in mortality at higher TCC levels, driven by a small subset of tracts, may reflect the underlying demographic composition of older populations in high‐canopy areas, rather than a direct adverse effect of canopy cover itself.

Our interaction models suggest that the quality and maintenance of tree canopy may be even more important to consider than increasing tree planting. For clarity, we use tree maintenance to mean active arboricultural care (pruning, watering, mulching, pest/disease management) intended to sustain tree health and safety. We use maintaining the urban canopy to mean the broader objective of sustaining or increasing canopy cover over time, which depends on both canopy protection/retention (preventing avoidable removal or damage, including during development) and ongoing maintenance (Vogt et al., 2015). Previous work has found that the placement and maintenance of trees may enhance their cooling benefits, which can drive reductions in heat‐related stress and mortality (Chicago Urban Forest Management Plan, 2023). Use of NLCD data in our study showed a decline in tree canopy over time, which is consistent with reports of TCC loss in Cook County despite broader gains in surrounding counties (Chicago Urban Forest Management Plan, 2023). Although the net change over time has been small, our interaction plots show that greater yearly declines in TCC compound increases in mortality among census tracts experiencing the highest temperatures. The 2023 Chicago Urban Forests Management plan notes historical inequities in the city's response to resident requests to maintain urban tree canopy, with more resources allocated to neighborhoods on the North Side of the city, such as the community areas that comprise Cluster 1. Maintaining tree canopy over time may increase health benefits, even in areas with relatively few trees. For example, in Cluster 4 neighborhoods on the South Side, with similar absolute TCC to those in Cluster 1 on the North Side, the lack of canopy maintenance may be worsening year‐to‐year TCC decline and contributing to higher mortality rates (Chicago Urban Forest Management Plan, 2023). Community‐supported watering and other grassroots maintenance initiatives may ensure trees are resilient to climate change and maximize their cooling benefits (“Chicago Region Trees Initiative”, 2024).

Previous evaluations of tree canopy and mortality in Chicago have yielded mixed findings, with some studies finding significant associations between cross‐sectional measurements of tree canopy and reductions in mortality (Sinha et al., 2022) whereas others have not (Folkmann et al., 2024). A significant challenge in studying tree canopy changes lies in the availability and structure of data sets. Many studies rely on Light Detection and Ranging (LiDAR) for precise canopy measurement, yet year‐to‐year LiDAR data are scarce, limiting longitudinal analysis (Astell‐Burt & Feng, 2020; Folkmann et al., 2024; Keralis et al., 2020; Lovasi et al., 2008; Sinha et al., 2022; Ulmer et al., 2016; Wei et al., 2024). Other studies use measures such as the NDVI, which captures general greenness but does not exclusively reflect tree canopy (Astell‐Burt & Feng, 2020; Keralis et al., 2020; Kim et al., 2024; Knobel et al., 2021; Wilker et al., 2014). Unlike NDVI, which represents a continuous measure of greenness, tree canopy represents a discrete element that can be directly modified by public interventions. This distinction is essential for interpreting health impacts, as urban planning initiatives in Chicago and other cities globally more often involve policies aimed at increasing canopy cover rather than overall greenness (“Chicago Region Trees Initiative”, 2024; Mcdonald et al., 2024). Indeed, a previous longitudinal study in Portland, Oregon found similar patterns to those we model: areas with more tree planting had less mortality (Donovan et al., 2022).

The stronger associations with mortality observed for year‐to‐year TCC change compared with annual TCC levels suggest that the health benefits of TCC may be most closely linked to sustained canopy expansion and maintenance, rather than static differences in baseline cover. These implications are particularly salient in the context of the recent termination of a $75 million U.S. Forest Service urban forestry grant program, which may shift the near‐term burden of implementation locally (Tesfaye, 2025). From a policy perspective, prioritizing interventions in neglected neighborhoods to prevent further TCC loss, such as targeted planting, long‐term maintenance budgets, and broad community partnership may best help sustain urban trees.

This study has several limitations. Our analysis is associative in nature, and we were unable to directly establish causality. Previous studies that have explored causal relationships have relied on participant‐level data, such as long‐term exposure to tree canopy. We were unable to ascertain how many years a person had lived at the address listed on their death record, limiting our ability to explore individual‐level exposure over time. The data sets used in this study also pose certain limitations, particularly in terms of spatial resolution. LiDAR, which has been used by the City of Chicago and the Morton Arboretum for its tree census, provides high‐resolution 4‐m tree canopy data and is therefore more equipped to detect newly planted smaller canopied trees compared to the 30‐m resolution NLCD data used in this study (“Chicago Region Trees Initiative”, 2024). Inability to detect smaller trees may also explain the lower absolute TCC percent estimates in NLCD maps, as compared to estimates from the Morton Arboretum. We used USDA Forest Service data because it is publicly available, widely utilized in urban forestry research, and allowed for longitudinal analyses. While NLCD TCC data may be unable to resolve small canopied trees we argue that it is sufficient to capture most larger established and health‐protective trees as well as broad year‐to‐year TCC change. (“Chicago Region Trees Initiative”, 2024; Chicago Urban Forest Management Plan, 2023).

Our sensitivity analysis using Google TES‐based cross‐sectional analysis showed a small positive statistically significant effect, in contrast to our main results using NLCD data. Restricting to pre‐COVID years using NDVI and NLCD data demonstrated a persistent statistically significant protective effect for year‐to‐year changes (Supporting Information S1). This may be due to limitations of the TES data resolution, which may not capture smaller, newly planted trees, leading to lower reported TCC among higher income neighborhoods in the North Chicago region. TES data also only reflects canopy from a single year, whereas the longitudinal NLCD and NDVI data sets capture year‐to‐year variation and provides a more robust assessment of long‐term trends. Daymet temperature estimates are spatially comprehensive and assimilate National Oceanic and Atmospheric Administration (NOAA) weather station observations from point locales. Although Daymet estimates do not explicitly take local TCC into account, they are indirectly influenced by factors which impact NOAA station measures, such as local land cover and urban heat island effects. Daymet also may smooth localized temperature extremes due to its gridding algorithm and spatial resolution. However, its high spatial resolution facilitates a more granular analysis consistent with available demographic data, while its public availability enhances reproducibility. Although we analyzed temperature as a potential effect measure modifier of the canopy‐mortality association in this study, temperature may also be conceptualized as a mediator along the canopy‐mortality causal pathway. A formal mediation analysis of this association could serve as the subject of future research. Though we controlled for air pollution as a confounder of the tree canopy and mortality association, there is likely a bidirectional relationship between tree canopy and air pollution, which is unaccounted for in this study (Markevych et al., 2017). Finally, although we included Chicago region in our models to control for unobserved area‐level confounding, such as sociodemographic population and policy shifts over time, full control of all confounders of the canopy‐mortality association may have been incomplete. Despite these constraints, our findings provide valuable insights into the role of urban tree canopy in shaping population health outcomes, underscore the need for continued investment in urban tree planting and maintenance as a public health strategy, and support further work investigating causal mechanisms (Bhatnagar et al., 2024).

## 5 Conclusion

This study represents one of the first longitudinal investigations of year‐to‐year changes in TCC and its association with mortality outcomes, demonstrating how trees can relate to health outcomes over time. Our spatial analysis reveals the clustering of persistent health disparities: neighborhoods on the South and West Sides of Chicago continue to experience elevated mortality rates despite higher‐than‐average TCC, underscoring the complexity of contributing factors such as heat stress, canopy loss over time due to poor maintenance, and socioeconomic inequities. Addressing these disparities effectively will likely require developing targeted public health interventions and equitable urban planning.

Our study suggests that expanding, and even more importantly, maintaining tree canopy in areas affected by high summer temperatures presents a key opportunity to enhance public health, mitigate climate‐related impacts, and potentially reduce health inequities, including death from a broad range of associated cardiovascular, respiratory, musculoskeletal and mental health/psychiatric conditions. Increased funding for urban tree planting and maintenance could facilitate the prioritization of initiatives that benefit underserved communities, advancing both environmental justice and overall health equity in urban environments. Future studies should employ similar methods at a greater geographic scale to identify populations most vulnerable to heat stress and effectively target tree canopy interventions.

## Conflict of Interest

The authors declare no conflicts of interest relevant to this study.

## Supporting information

Supporting Information S1

## Data Availability

The data and code supporting the findings of this study are openly available in Prism (Garcia et al., 2026). The repository contains processed census tract‐level NLCD TCC (Dewitz, 2023) and NDVI (Vermote, 2019) data for Chicago (2011–2021), processed tract‐level max summer temperature (Thornton et al., 2022) and NO_2_ air quality data (Anenberg et al., 2022), along with scripts used for data processing and aggregation. The mortality data used in this study were obtained from the Illinois and Chicago Departments of Public Health. These records contain sensitive information and are not publicly available due to privacy protections. Researchers registered with the Illinois and Chicago Departments of Public Health may access these records subject to applicable data governance and confidentiality requirements.
