# Supplementary material for: Increasing Urban Tree Canopy Associated With Reduced Mortality: A Longitudinal Analysis of Chicago Neighborhoods
Source: Geohealth. 2026 Aug 5;10(8):e2025GH001651. doi: 10.1029/2025GH001651 (PMC13440163; doi:10.1029/2025GH001651)
Supplement: Supplementary file 1 [file GH2-10-e2025GH001651-s001.pdf]

GeoHealth

Supporting Information for

Increasing urban tree canopy associated with reduced mortality: a longitudinal analysis of Chicago neighborhoods

Harrison C. Garcia[1,2], Peter M. Graffy[1,2,3,4], Benjamin W. Barrett[1,2,3,4], Maxime A. Visa[1,2], Jenny Jia[1,5,6], Evan Mallen[7], Raed Mansour[2,8], Graham Briggs[9], Trent Ford[10], Donald J. Wuebbles[11], Norrina Allen[1,2,4,5], Teresa H. Horton[2,12], Abel Kho[1,2,4,5], & Daniel E. Horton[2,13]

[1] Feinberg School of Medicine, Northwestern University, Chicago, IL USA

[2] Defusing Disaster Working Group, Buffett Institute for Global Affairs, Northwestern University, Evanston, IL, USA

[3] Center for Education in Health Sciences, Northwestern University, Chicago, IL USA

[4] Department of Preventive Medicine, Northwestern University, Chicago, IL USA

[5] Institute for Public Health and Medicine, Northwestern University, Chicago, IL USA

[6] Clinical and Translational Sciences Institute, Northwestern University, Chicago, IL USA

[7] Urban Climate Lab, Georgia Institute of Technology, Atlanta, GA USA

[8] Metropolitan Planning Council, Chicago, IL USA

[9] Illinois Department of Public Health, Chicago, IL USA

[10] Prairie Research Institute, University of Illinois Urbana-Champaign, Champaign, IL USA

[11] Department of Climate, Meteorology, and Atmospheric Sciences, University of Illinois Urbana-Champaign, Urbana, IL USA

[12] Department of Anthropology, Northwestern University, Evanston, IL USA

[13] Department of Earth, Environmental, and Planetary Sciences, Northwestern University, Evanston, IL USA

Corresponding author: Harrison Garcia (harrison.garcia@northwestern.edu)

Contents of this file

Text for Sensitivity analysis
Supplemental Figure 1
Supplemental Table 1
Supplemental Figure 2
Supplemental Figure 3

Introduction

This supplemental file contains detailed methods and results of our sensitivity analysis, as well as a supplemental figure of Quintiles of tree canopy percent land area coverage by community area, a supplemental table of counts of ICD-10 CM code prefixes associated with Chicago mortality records, as well as supplemental figures to better contextualize the results of our canopy-temperature interaction plots.

Sensitivity Analysis

We ran three sensitivity analyses including and excluding COVID-era years (restricted to pre-2020) in the same negative binomial GEE framework for various greenness and tree data sources: 1) refit NLCD total TCC and year-to-year TCC change models (Dewitz, 2023) 2) substituted USDA canopy with one year of Google Tree Equity Score (TES) canopy (*Tree Equity Score: Methods and Data [Dataset]*, 2024), and 3) substituted USDA canopy with annual tract-level normalized difference vegetation index (NDVI) from 2011-2021 along with year-to-year changes (Vermote, 2019). The USDA NLCD dataset was chosen as the primary source because it provides annual tree canopy maps from 2011 to 2021, allowing for longitudinal analysis of changes in canopy cover over time. In contrast, the Google TES dataset represents only a single time point around 2020, lacking the temporality needed to assess year-over-year changes in canopy coverage. However, the Google dataset has a higher spatial resolution (1-meter) compared to the USDA's 30-meter resolution, which may allow for more precise local canopy estimates. NDVI is computed annually and is one of the most widely used nature-exposure metrics in epidemiologic studies. NDVI effects are reported per 0.1 unit increase.

In contrast to the main analysis, pre-COVID models showed a statistically significant positive association for total TCC, with higher average NLCD TCC associated with increased mortality (IRR = 1.016, 95% CI: 1.008–1.025). The direction and magnitude of associations for pre-COVID year-to-year increases in TCC remained consistent with the main analysis, with greater annual canopy gains significantly associated with lower mortality (IRR = 0.902, 95% CI: 0.867–0.938).

When USDA NLCD canopy was replaced with Google Tree Equity Score canopy, a slight but statistically significant positive association with mortality was noted (IRR = 1.006, 95% CI: 1.002–1.011).

In NDVI-based sensitivity analyses, neighborhood greenness levels (IRR = 1.003, 95% CI: 1.000–1.006) and year-to-year changes (IRR = 0.998, 95% CI: 0.995–1.000) were associated with mortality but with very small effect sizes in models including all study years. When analyses were restricted to pre-COVID years, total NDVI had a statistically significant positive association with mortality (IRR = 1.006, 95% CI: 1.003–1.010). In contrast, year-to-year increases in NDVI were statistically significantly protective (IRR = 0.997, 95% CI: 0.994–0.999).

The similar direction and statistical significance of year-to-year change in NLCD and NDVI TCC in our pre-COVID models indicates that our main conclusions were not driven by pandemic-era deaths. Prior studies in Chicago using Google-derived tree canopy data have shown protective effects, contrasting with our findings (Stuhlmacher & Kim, 2024). However, our study revealed clusters of neighborhoods on the South and West sides characterized by increased mortality, higher TCC, and hotter temperatures. Annual TCC from NLCD and Google TES estimates being associated with increased mortality points to potential unmeasured confounding by broader sociodemographic and built environment factors. Our included controls, such as air quality, income, Shannon racial/ethnic entropy, and Chicago region likely only partially capture confounders affecting the relationship between TCC and mortality. Factors such as proximity to the lake and broader policy changes may explain the positive association between TCC and mortality. However, our models showed that increasing TCC over time is protective.

References

Dewitz, J. (2023). *National Land Cover Database (NLCD) 2021 Products: U.S. Geological Survey data release [Dataset]*. Retrieved from: https://doi.org/10.5066/P9JZ7AO3

Stuhlmacher, M., & Kim, Y. (2024). The unequal connectivity of parks and tree canopy in 10 US cities. *Urban Forestry & Urban Greening, 96*, 128332. https://doi.org/10.1016/j.ufug.2024.128332

Tree Equity Score: Methods and Data [Dataset]. (2024). Retrieved from: https://www.treeequityscore.org/methodology

Vermote, E. (2019). NOAA climate data record (CDR) of AVHRR normalized difference vegetation index (NDVI), version 5 [Dataset]. *NOAA National Centers for Environmental Information, 10*, V5ZG6QH9. https://doi.org/10.7289/V5ZG6QH9

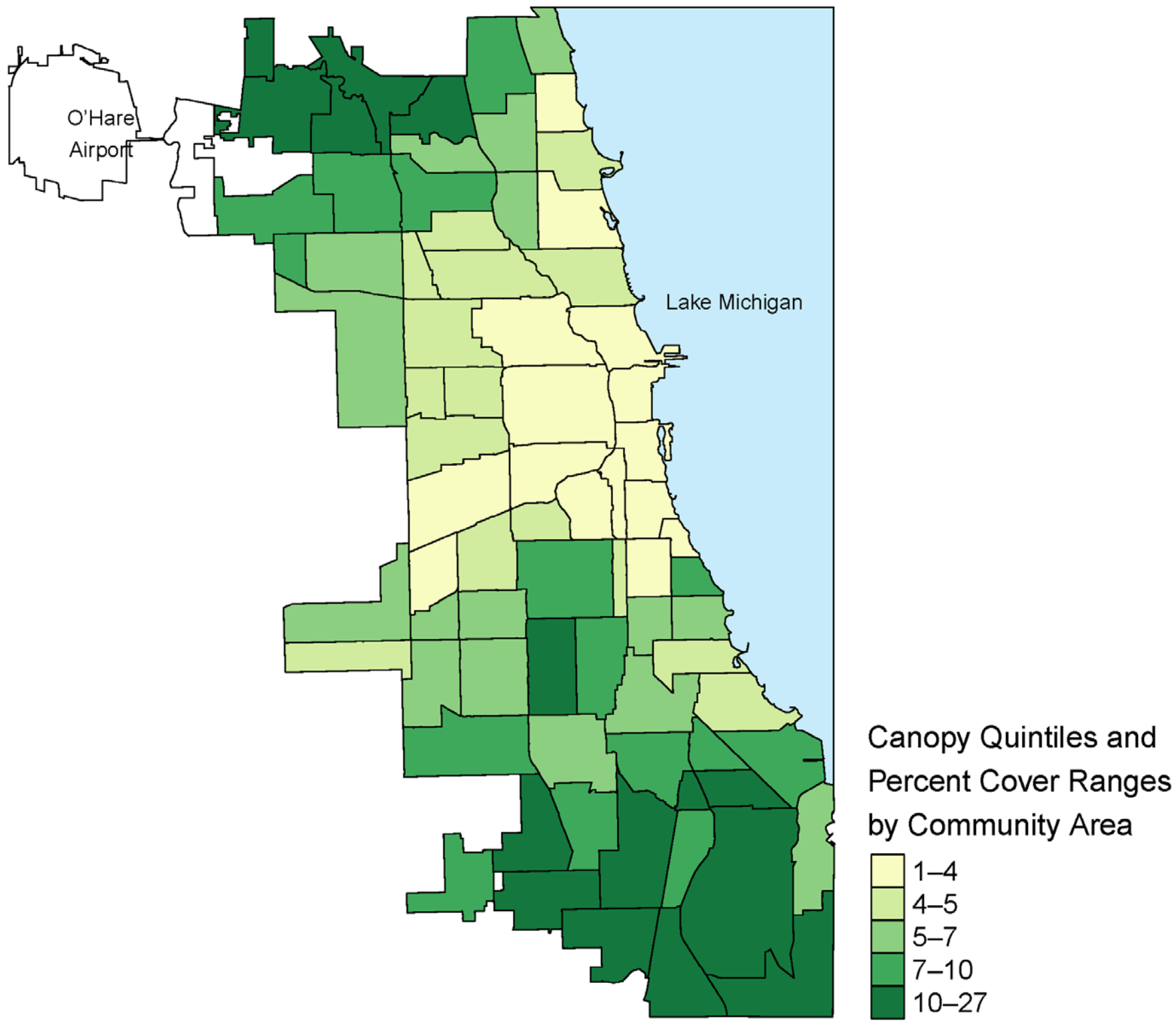

Supplemental Figure 1: Quintiles of tree canopy percent land area coverage by community area (averaged over 2011-2021)

Category	Subcategory	ICD-10 Prefix	Count
Cardiovascular	Ischemic Heart Diseases	I20–I25	47055
	Hypertensive Diseases	I10–I15	38132
	Heart Failure	I50	27809
	Other Heart Diseases	I30–I52	26728
	Cerebrovascular Diseases	I60–I69	18745
	Diseases of Arteries	I70–I79	5031
	Pulmonary Heart Disease	I26–I28	2993
	Diseases of Veins, Lymphatics	I80–I89	1657
	Other Circulatory Diseases	I95–I99	1349
	Rheumatic Heart Disease	I05–I09	352
Mental Health/Psychiatric	Substance Use Disorders	F10–F19	22053
	Organic Disorders	F00–F09	14022
	Mood Disorders	F30–F39	479
	Schizophrenia and Related Disorders	F20–F29	318
	Neurotic and Stress-Related Disorders	F40–F48	118
	Intellectual Disabilities	F70–F79	80
	Behavioral Syndromes	F50–F59	68
	Childhood Behavioral Disorders	F90–F98	46
	Psychological Development Disorders	F80–F89	33
	Personality Disorders	F60–F69	4
Musculoskeletal	Dorsopathies	M40–M54	604
	Osteoarthritis	M15–M19	514
	Inflammatory Polyarthropathies	M05–M14	378
	Soft Tissue Disorders	M60–M79	287
	Other Joint Disorders	M20–M25	51
	Bone Disorders	M80–M99	47
Respiratory	Lung Diseases Due to External Agents	J60–J70	15805
	Influenza and Pneumonia	J09–J18	15600
	Chronic Lower Respiratory Diseases	J40–J47	111
	Acute Upper Respiratory Infections	J00–J06	44
	Other Acute Lower Respiratory Infections	J20–J22	24

Supplemental Table 1: Counts were derived by tabulating all mortality-associated ICD-10 codes from prefixes listed in the Chicago vital records. These were used to identify deaths eligible for inclusion in the disease-specific mortality analysis. Because decedents may have multiple listed causes/conditions, categories are not mutually exclusive and individuals may be counted in more than one ICD subset. As a result, summed counts can exceed the total number of decedents.

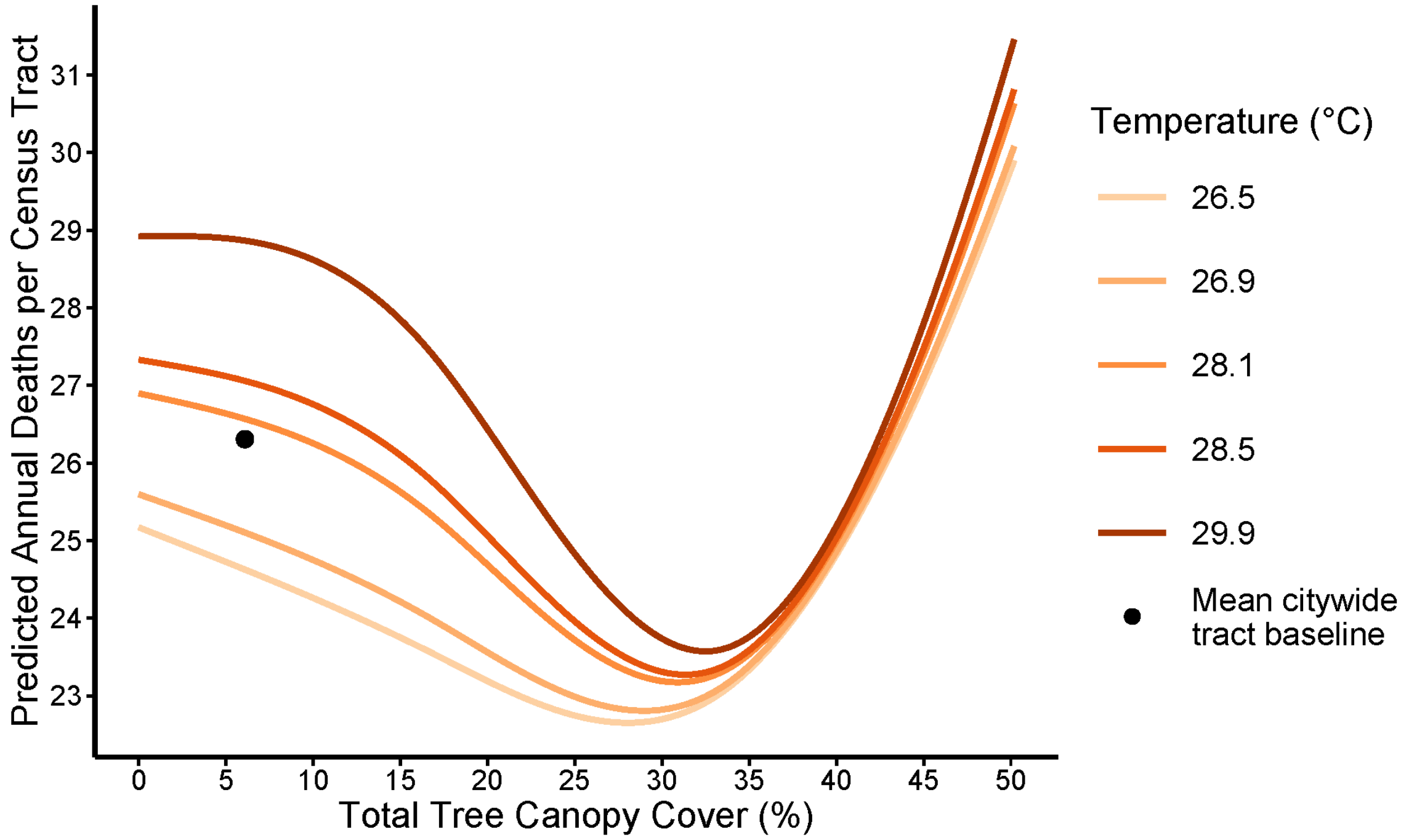

Supplemental Figure 2: Spline-based interaction effects of tree canopy cover and temperature on mortality. Estimates were derived from negative binomial generalized estimating equation models using natural spline terms for tree canopy cover, adjusting for census tract–level average maximum summer temperature, median income, ethnic/racial entropy, NO_2 concentrations, Chicago region, and a population log offset. The model displayed uses a spline specification with three degrees of freedom, which provided the best fit compared four and five degrees of freedom models, as determined by QIC from the MuMIn package.

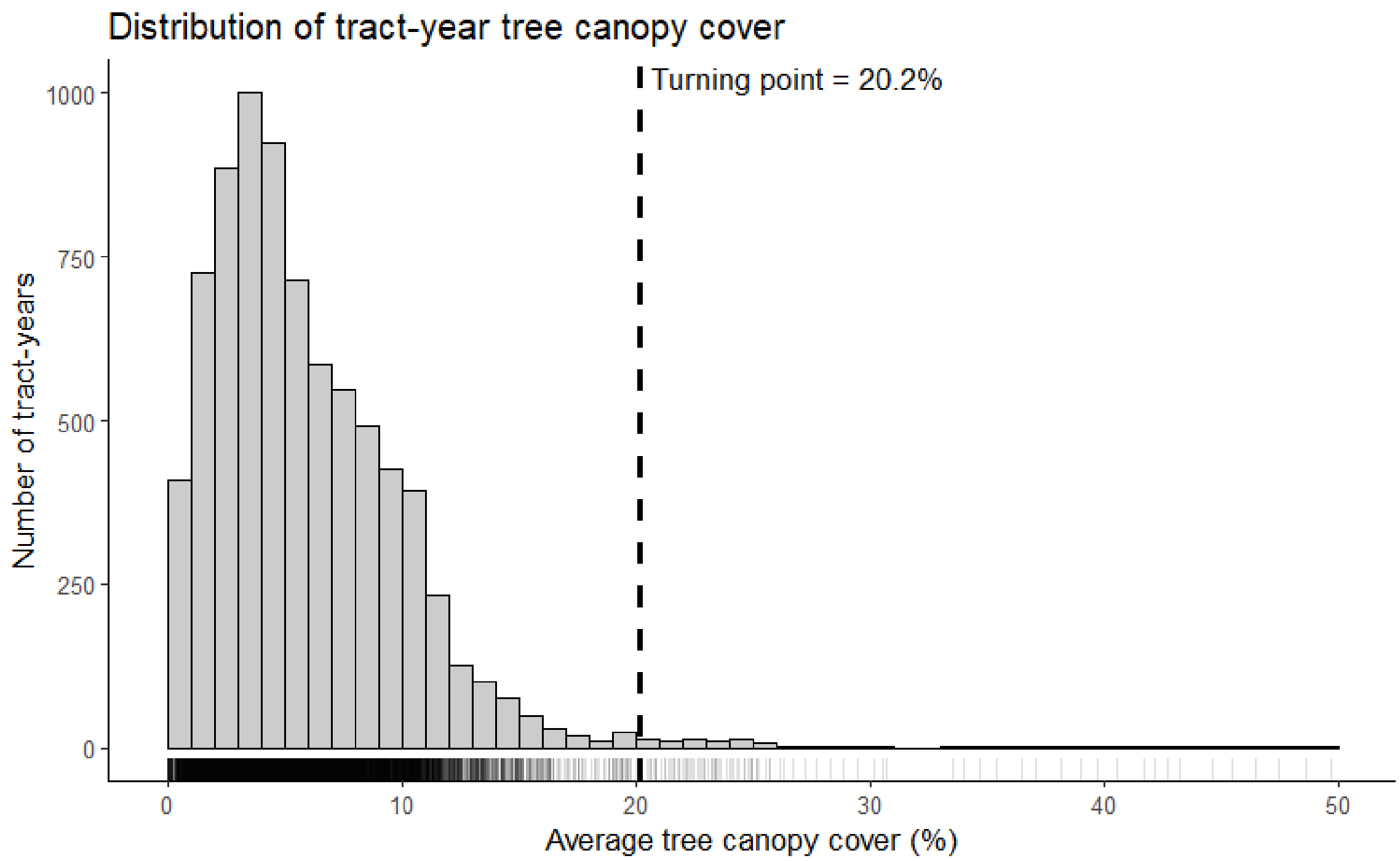

Supplemental Figure 3: Distribution of total tree canopy cover (TCC) across tract-years. Histogram of TCC (%) with tract-year counts on the y-axis. The reference value of 20.2% corresponds to the mean TCC of Cluster 3, derived from the clustering analysis of community areas. This TCC value also corresponds to the turning point in **Figure 2a** where mortality was predicted to increase as TCC further increased from this value, although the model used to derive this figure was not statistically significant.
